# 

**Published:** 1792

**Authors:** 


### MEDICAL FACTS

AND

### OBSERVATIONS.

VOLUME THE SECOND.

Surgeon Genl's Office
LIBRARY.
Washington D.C.

LONDON:

PRINTED FOR J. JOHNSON, N° 72, ST. PAUL'S CHURCH YARD.

M.DCC.XCII.

DIRECTIONS TO THE BINDER.

Plate the Firſt may be placed at page 18, where the references to it are explained; and Plate the Second at page 162.

MEDICAL

be neceſſary, our author thinks, to prepare it by means of manganeſe that has been previouſly ſeparated, by a chemical proceſs, from the lead and other metals with which that ſubſtance is uſually contaminated.

---

### CATALOGUE OF BOOKS.

1. THE Chirurgical Works of *Percivall Pott*, F. R. S. Surgeon to St. Bartholomew's Hoſpital. A new Edition, with his laſt Corrections. To which are added, a ſhort Account of the Life of the Author, a Method of curing the Hydrocele by Injection, and occaſional Notes and Obſervations. By *James Earle*, Eſq. Surgeon Extraordinary to His Majeſty's Houſehold, and Surgeon to St. Bartholomew's Hoſpital. 3 Vols. 8vo. *Johnſon*, London, 1790.

2. An hiſtorical Inveſtigation into the firſt Appearance of the Venereal Diſeaſe in Europe; with Remarks on its particular Nature. To which are added, Obſervations on the Non-neceſſity of Quarantines being obſerved againſt the the Plague by the Veſſels arriving from the Mediterranean at Britiſh, French, or other Ports. By M. *Sanchéz*, Doctor of the Faculty of Medicine at Paris. Tranſlated from the French by *Joſeph Skinner*, Surgeon, Tranſlator of Fontana on Poiſons, &c. 8vo. *Johnſon*, London, 1790.

3. The Apothecary's Mirror; or, The preſent State of Pharmacy exploded; in a Letter to *J. H. Sequeira*, M. D. By Diſcriminator. 12mo. *Macrae*, London, 1790.

4. Chemical Experiments and Opinions; extracted from a Work publiſhed in the laſt Century. 8vo. *Murray*, London, 1790.

5. A Philoſophical Inquiry into the Nature and Properties of Common Water. By *Polydore Lewis*, M. D. 8vo. *Walter*, London, 1790.

6. Eſſays on Fractures and Luxations. By *John Aitken*, M. D. Fellow of the Royal College of Surgeons, &c. Illuſtrated with eleven Plates. 8vo. *Cadell*, London, 1790.

7. A Treatiſe on the Strangles and Fevers of Horſes: with a Plate repreſenting a Horſe in the Staggers, ſlung. By *Thomas Proſſer*. 8vo. *Grant*, London, 1790.

8. Annual Oration, delivered March 8th, 1790, before the Medical Society, Bolt Court, Fleet

Fleet Street, London, by *George Wallis*, M. D. one of the Fellows, and Lecturer on the Theory and Practice of Phyfic. 4to. *Robinfons*, London, 1790.

9. A Treatife on One Hundred and Eighteen Difeafes of the Eyes and Eyelids; in which are communicated feveral new Difcoveries relative to the Cure of Defects in Vifion; with original Prefcriptions. By *William Rowley*, M. D. Member of the Univerfity of Oxford, the Royal College of Phyficians in London, &c. 8vo. *E. Newbery*, London, 1790.

10. Chemical and Economical Effays, defigned to illuftrate the Connexion between the Theory and Practice of Chemiftry, and the Application of that Science to fome of the Arts and Manufactures of the United States of America. By *John Pennington*. 8vo. Philadelphia, 1790.

11. The Defcriptions and Characters of the different Difeafes of the Human Body; to which is added, an Arrangement of the Medicines and Preparations, in the London Pharmacopœia, according to their refpective Virtues: Being the firft Volume of the Franklinean Improvement of Medicine, or an Attempt, according to Dr. Franklin's Predictions, to to establish the Means of rendering Sickness and Disease less injurious, dangerous, and fatal to Health, and thus of causing old Age to be again natural to Man. By *George Edwards*, Esq. M. D. Author of the Aggrandisement of Great Britain, &c. 4to. *Ridgway*, London, 1791.

12. Cautions to the Heads of Families, in three Essays: 1. On Cyder Wine, prepared in Copper Vessels; with Hints for the Improvement of Cyder, Perry, and other Fruit Liquors: 2. On the Poison of Lead—Method of detecting it in various Liquors, Foods, Medicines, Cosmeticks, &c., with general Indications of Cure: 3. On the Poison of Copper—How it may be discovered, though in very minute Quantity — Method of Cure. By *A. Fothergill*, M. D. F. R. S. Member of the Royal College of Physicians, London, and of the Medical Societies of London, Edinburgh, and Paris. 8vo. Bath, 1791.

13. An Essay on the injurious Custom of Mothers not suckling their own Children; with some Directions for chusing a Nurse, and weaning of Children, &c. By *Benjamin Lara*, Surgeon, Member of the Corporation of Surgeons geons in London, and Practitioner in Midwifery. 12mo. *Moore*, London, 1791.

14. Elements of Chemiſtry. By M. *J. A. Chaptal*, Chevalier of the Order of the King, Profeſſor of Chemiſtry at Montpellier, Honorary Inſpector of the Mines of France, and Member of ſeveral Academies of Sciences, Medicine, Agriculture, Inſcriptions, and Belles Lettres. Tranſlated from the French, by *William Nicholſon*, Author of the Firſt Principles of Chemiſtry, &c. 3 Vols. 8vo. *Robinſons*, London, 1791.

15. An Inquiry into the Cauſes which produce, and the Means of preventing, Diſeaſes among Britiſh Officers, Soldiers, and others, in the Weſt Indies: Containing Obſervations on the Mode of Action of ſpirituous Liquors on the Human Body; on the Uſe of Malt Liquors, and on ſalted Proviſions; with Remarks on the moſt proper Means of preſerving them: Alſo Notes relating to ſome Particulars in the Britiſh Army in Ireland and the Weſt Indies. By *John Bell*, M. D. Member of the Royal Medical Society of Edinburgh, Phyſician in London, and formerly Surgeon to the late ninety-fourth and to the fifth (or Northumberland)

land) Regiment of Foot. 8vo. *Murray*, London, 1791.

16. Defcription of a portable Cheft of Chemiftry; or, Complete Collection of chemical Tefts, for the Ufe of Chemifts, Phyficians, Mineralogifts, Metallurgifts, fcientific Artifts, Manufacturers, Farmers, and the Cultivators of natural Philofophy: Invented by *J. F. A. Gottling*, Profeffor of Chemiftry at Jena in Saxony. Tranflated from the original German. 8vo. *Kearfley*, London, 1791.

17. An Effay on the Vitality of the Blood. By *James Corrie*, M. D. 8vo. *Elliot* and *Kay*, London, 1791.

18. A Treatife on the Diagnofis and Prognofis of Difeafes. Part I. Containing an Hiftory of internal phlegmonous Inflammation. By *Philip Parry Price*. 8vo. *Johnfon*, London, 1791.

19. A Differtation on Gonorrhœa, and fome other Effects of the venereal Virus. By *Edward Collis*, F. A. S. S. Honorary Prefident of the Hibernian Medical Society, &c. 12mo. Edinburgh, 1791.

20. Obfervations on the Small Pox and Inoculation. To which is prefixed, a Criticifm upon Dr. *Robert Walker*'s late Publication on the the Subject. By *Alexander Aberdour*, Surgeon in Alloa. 8vo. Edinburgh, 1791.

21. A Differtation on fufpended Refpiration from Drowning, Hanging, and Suffocation; in which is recommended a different Mode of Treatment to any hitherto pointed out. By *Edward Coleman*, Surgeon. 8vo. *Johnfon*, London, 1791.

22. A Treatife on the Origin and component Parts of the Stone in the urinary Bladder, being the Subftance of the Gulftonian Lectures read at the College of Phyficians in the Year 1790. By *William Auftin*, M. D. Fellow of the College of Phyficians, and Phyfician to St. Bartholomew's Hofpital. 8vo. *Nicol*, London, 1791.

23. A Lecture on Mufcular Motion, read at the Royal Society the 13th and 20th of November, 1788. By *Gilbert Blane*, M. D. F. R. S. 4to. *Murray*, London, 1791.

24. A Treatife on the Hydrocele: Containing an Examination of all the ufual Methods of obtaining Relief in that Difeafe. The radical Cure by Injection is particularly defcribed, and illuftrated with Cafes. By *James Earle*, Efq. Surgeon Extraordinary to His Majefty's Houfehold, and fenior Surgeon to St. Bartholomew's lomew's Hofpital. 8vo. *Johnfon*, London, 1791.

25. A Treatife on the Digeftion of Food. By *G. Fordyce*, M. D. F. R. S. Fellow of the Royal College of Phyficians, and Reader on the Practice of Phyfic in London. 8vo. *Johnfon*, London, 1791.

26. An Effay on vital Sufpenfion; being an Attempt to inveftigate and to afcertain thofe Difeafes in which the Principles of Life are apparently extinguifhed. By a Medical Practitioner. 8vo. *Rivingtons*, London, 1791.

27. An Expofition of the Principles of Anatomy and Phyfiology; containing the Prælectiones Anatomicæ of *Ferdinand Leber*, tranflated from the Original, publifhed in Latin at Vienna. By *Walter Vaughan*, M. D. 2 Vols. 8vo. *Robinfons*, London, 1791.

28. The Utility of Medical Electricity illuftrated, in a Series of Cafes and practical Obfervations; tending to prove the Superiority of Vibrations to every other Mode of applying the electric Fluid. By *Francis Lowndes*. 8vo. *Johnfon*, London, 1791.

29. A fhort Inquiry into the Merits of a new-difcovered Fact, of a relative Nature, in the venereal Poifon. By *Thomas Ogle*, jun.

 Surgeon

Surgeon Extraordinary to His Royal Highneſs the Prince of Wales. 8vo. *Johnſon*, London, 1791.

30. An Inquiry into the Effects of ſpirituous Liquors upon the Human Body, and their Influence upon the Happineſs of Society *. By *Benjamin Ruſh*, M. D. Profeſſor of the Theory and Practice of Medicine in the College of Philadelphia. The third Edition. 8vo. Philadelphia, 1791.

31. An Inaugural Diſſertation on univerſal Dropſy. By *Elijah Perkins*, A. B. of Connecticut. 8vo. Philadelphia, 1791.

32. An Inaugural Diſſertation on the chemical Properties of atmoſpheric Air. By *William R. Cozens*, of New Jerſey. 8vo. Philadelphia, 1791.

33. An Inaugural Diſſertation on Cholera Morbus. By *David Hoſſack*, A. B. of New York. 8vo. Philadelphia, 1791.

* This pamphlet is accompanied with "A moral and phy-"ſical Thermometer; or, A Scale of the Progreſs of Tem-"perance and Intemperance;" in which the effects of different liquors are exhibited, at one view, in their proper order. This was originally publiſhed, by Dr. Ruſh, in the Columbian Magazine for January, 1789.

34. Corre-

34. Correſpondence for the Introduction of Cochineal Inſects from America, the Varniſh and Tallow Trees from China, the Diſcovery and Culture of white Lac, the Culture of red Lac, and alſo for the Introduction, Culture, and Eſtabliſhment of Mulberry Trees and Silk Worms; with a Deſcription and Drawing of an improved Piemonteſe Reel for the Manufacture of raw Silk; together with the Culture of the fineſt Cinnamon Trees of Ceylon, Indigo, and ſome other valuable Articles. By *James Anderſon*, M. D. 8vo. Madras, 1791.

35. Aphroſidiacus, ſive de Lue Venerea, in duas Partes diviſus, quarum altera continet ejus Veſtigia in veterum Auctorum Monimentis obvia, altera quos *Aloyſius Luiſinus* temere omiſit Scriptores, et medicos et hiſtoricos, Ordine chronologico digeſtos. Collegit, Notulis inſtruxit, Gloſſarium, Indicemque Rerum memorabilium ſubjicit, *C. G. Gruner*, Med. D. et Prof. &c. Folio. Jena, 1789.

36. Diſſertatio Inauguralis Medica, qua diſquiritur, an Stimuli morboſi quandoque ſileant; Præſide *G. G. Ploucquet*, Auctore *J. C. Knapp*, Wirtembergico-Einſidelenſe. 4to. Tubingen, 1789.

Medicamentis vomitoriis; Auctore *Samuele Burton Pearson*, Anglo. 8vo. Edin. 1790.

54. Disputatio Medica Inauguralis quædam de Hysteria complectens; Auctore *Gulielmo Robertson*, Scoto. 8vo. Edin. 1790.

55. Tentamen Medicum Inaugurale de Dysenteria; Auctore *Joanne Starrat*, Hiberno. 8vo. Edin. 1790.

56. Tentamen Medicum Inaugurale de Cynanche maligna; Auctore *Thoma Wilson*, Hiberno. 8vo. Edin. 1790.

57. Dissertatio Medica Inauguralis de Variolarum Contagionis Actione; Auctore *Jacobo Clidsdale*, Scoto. 4to. Lugduni Batavorum, 1790.

58. Dissertatio Inauguralis Medica de Lysimachiæ purpureæ, sive Lythri Salicariæ *Linn.* Virtute medicinali non dubia; Auctore *Joanne Scherbio*, Moeno-Francofurtano. 4to. Jenæ, 1790.

59. Dissertatio Inauguralis Medica de Digitali purpurea ejusque Usu in Scrofulis medico; Auctore *Joanne Jacobo Merz*, Hammelburgo-Fuldensi. 4to. Jenæ, 1790.

60. D. *Samuelis Gottl. Vogelii*, Seren. Duc. regn. Megapol. a Conf. aul. P. p. o. in Univers. litt. Rostoch. Diatribe Medico-politica de Causis quare quare tot Submerſi in Vitam non revocentur. 8vo. Hamburgi, 1790.

61. Regimen Sanitatis Salerni, ſive Scholæ Salernitanæ de conſervanda bona Valetudine Præcepta. Edidit, Studii medici Salernitani Hiſtoria præmiſſa, *Jo Chr. Gottl. Ackermann*, M. D. & in Univ. litt. Altorſina P. p. o. 8vo. Stendal, 1790.

62. Prælectio Academica, ſimplitiores & ſalubriores comprehendens de Febribus Notiones, elucubrata a *Joſ. Pinille Vizayno*, defenſanda a *J. R. Baquero*. 4to. Alcala de Henares, 1790.

63. Delectus Opuſculorum ad Scientiam naturalem ſpectantium. Edidit *Chriſtianus Frider. Ludwig*, Hiſtoriæ Naturalis in Univ. Litt. Lipſ. Profeſſor. Volumen I. 8vo. Lipſiæ, 1790.

64. Opuſcula* Anatomica & Phyſiologica; retractata, aucta & reviſa ab Autore *Joh. Dan. Metzger*, S. R. M. Bor. Archiatro & Conſ. aul. Anat. & Med. Prof. prim. in Acad. Regiom. 8vo. Gotha, 1790.

* Viz. 1. Primi Paris Nervorum Hiſtoria; 2. Specimen Anatomes comparatæ primi Paris Nervorum; 3. Animadverſiones anatomico-phyſiologicæ in Doctrinam Nervorum.

65. Corona* Floræ Monspelienſis; Auctore *J. L. Victor Brouſſonet*, Monspeſſulano. 8vo. Monſpelii, 1790.

66. *Nat. Joſ. de Necker*, Botan. Sereniſſimi Electoris Bavaro-Palatini; Hiſtoriograph. utriuſque Ducat. Juliac. ac Montenſis; Acad. Scient. Mannh. Memb. ordinarii; Academiar. diverſ. Scient. Europæ Soc. extranei, Elementa Botanica, Genera genuina, Species naturales omnium Vegetabilium detectorum eorumque characteres diagnoſticos ac peculiares exhibentia, ſecundum Syſtema omologicum ſeu naturale, evulgata: cum Tabulis ſeparatis. 8vo. Tom. III. Neowedæ ad Rhenum, 1790.

67. Theſes Botanicæ in Uſum Auditorum Typis exſcriptæ Auctore *Paulo Dieterico Giſeke*, M. D. Phyſ. Prof. in Gymnaſ. Hamburgenſi & Bibliothecario ſecundo, Acad. Imp. Nat. Cur. Reg. Inſtit. Hiſt. Goett. Soc. Oecon. Lipſ. Sodali. 4to. Hamburgi, 1790.

68. Fungi Mecklenburgenſes ſelecti; Auctore *Henrico Julio Tode*, Synodi Wittenbur-

* "*Diſpoſitio operis*. 1. Incrementa Botanices Monſpelien-

"ſis; 2. Bibliotheca botanico-Monſpelienſis; 3. Plantæ di-

"catæ Botanicis Monſpeſſulanis; 4. Botanici Doctores Monſ-

"peſſulani; 5. Plantæ Monſpelienſes dictæ; 6. Plantæ olim

"Monſpelienſes."

genſis gensis præposito & V. D. apud Pritzierenses Ministro, Societ. Berol. Amic. Nat. Cur. nec non Nat. Cur. Halens. Sod. Faciculus I. nova Fungorum Genera complectens; Tabulis vii, æneis adjectis. 4to. Luneburgi, 1790.

69. Observations sur l'Efficacité du Melange d'Ether sulfurique et d'Huile volatile de Terebenthine, dans les Coliques hepatiques, produites par des Pierres biliaires; par M. *Durande*, Medecin des Etats de Bourgogne et de la Ville de Dijon, ancien Professeur de Chemie et de Botanique, Associé Regnicole de la Société Royale de Medecine, Agrégé honoraire au College Royal des Medecins de Nanci, des Academies de Dijon, de Montpellier, de Clermont. 8vo. Strasbourg, 1788.

70. Traité de la Dyssenterie; precedé d'un Memoire sur le Signe infaillible de la Mort; extrait des nouveaux Memoires de l'Academie Imperiale & Royale des Sciences & Belles Lettres de Bruxelles. Par M. *D****. 2 Tomes. 8vo. Bruxelles, 1789.

71. Methode nouvelle de traiter les Maladies Veneriennes, par les Gâteaux toniques Mercuriels, sans Clôture, & parmi les Troupes, sans sejour d'Hôpital, eprouvée dans les Ports du Roi: Ouvrage dans lequel on donne la

Compo-

Compofition des dits Gâteaux, ainfi que celle d'une Pommade particuliere; On y rend Compte de quelques Experiences eudiometriques: par M. *Bru*, Maitre en Chirurgie, ancien Chirurgien d'Armée & d'Infanterie, Chirurgien Major de la Marine, Directeur des Etabliffemens de Santé dans tous les Ports & Arfenaux du Roi, fous Lieutenant de la Garde Nationale Parifienne: Fait & publié par Ordre du Gouvernement, dedié a M. le Comte de *la Luzerne*, Miniftre de la Marine, approuvé par l'Academie Royale de Chirurgie. 2 Tomes. 8vo. Paris, 1789.

72. Memoire qui a remporté le Prix, en 1789, au Jugement de la Société Royale de Medecine de Paris fur la Queftion propofée en ces Termes: "Determiner, par l'obfervation, "qu'elles font les maladies qui refultent des "emanations des eaux ftagnantes, & des pays "marecageux, foit pour ceux qui habitent dans "les environs, foit pour ceux qui travaillent a "leur defféchement; & quels font les moyens "de les prevenir & d'y remedier;" par M. *Baumes*, Docteur en Medecine de l'Univerfité de Montpellier, Agrégé au College des Medecins de Nimes, Medecin de l'Hofpice de Charité de la même Ville, Associé Regnicole de la Société Royale de Medecine, &c. 8vo. Nimes, 1789.

73. Observations Chirurgico-legales, sur un Point important de la Jurisprudence criminelle, lues a la Seance publique de l'Acad. des Sciences de Dijon, le 20 Decembre, 1789, par le Professeur *Chaussier*. 8vo. Dijon, 1789.

74. Parallele entre les Misericordes et les Hopitaux. Par M. *D'Apples Gaulis*, du College de Medecine. 12mo. Lausanne, 1789.

75. Lettre de M. *Tissot*, a l'Auteur de la Gazette de Santé sur le Siege de la Pleuresie. 8vo. Lausanne, 1790.

76. Traité complet de la Petite Verole & de l'Inoculation, ou l'on fixe les vrais Principes de cette Maladie, & les Avantages de la nouvelle Methode curative très perfectionnée; avec des Observations, des Remarques & des Exemples pris dans differens Auteurs, & tirés d'une longue Experience de la Pratique des Inoculations. Ouvrage mis a la Portée de toutes sortes de Personnes, & dedié a M. *le Monnier*, premier Medecin du Roi. Par M. *Goetz*, Chevalier de l'Ordre du Roi, Docteur en Medecine, Inoculateur de Madame Elizabeth de France, Pensionnaire de LL. MM. le Roi de France & le Roi de Sardaigne, & Correspondant de l'Academie demie des Sciences de Turin. 8vo. Paris, 1790.

77. Elemens de Chimie, par M. *J. A. Chaptal*, Chevalier de l'Ordre du Roi, Profeſſeur de Chimie a Montpellier, Inſpecteur-honoraire des Mines du Royaume, & Membre de pluſieurs Academies des Sciences, de Medecine, d'Agriculture, d'Inſcriptions & belles Lettres. 3 Tomes. 8vo. Montpellier, 1790.

78. Deſcription des Plantes qui croiſſent aux Environs de Montauban, ou qu'on cultive dans les Jardins, rangées d'après la Methode ſexuelle avec l'Indication du Lieu ou elles viennent, & les Vertus principales des uſuelles. Par M. *Gaterau*, D. M. de Montpellier, & Membre du College de Medecine de Montauban. 8vo. Montauban, 1790.

79. Hiſtoire abregée de la Lithotomie, par M. *Saucerotte*, Maitre en Chirurgie gradué, Chirurgien ordinaire du feu Roi de Pologne, Staniſlas I. Aſſocié de l'Academie Royale de Chirurgie de Paris, l'un des Chirurgiens Majors du Corps ci devant de la Gendarmerie & actuellement des Carabiniers, Lithotomiſte penſionné pour la Lorraine & le Barrois. 8vo. Nanci, 1790.

80. Mo-

80. Memoire fur la Maniere dont fe forment les Pierres dans le Corps humain, & fur les Moyens de les diffoudre; par M. *L'Eftrade*, Medecin du Roi à Saint Pierre, Ile Martinique; de la Société Royale des Sciences & des Arts du Cap-Francois. 8vo. Martinico, 1790.

81. Catalogue methodique & raifonné de la Collection des Foffiles de Mlle *Eleonore de Raab*. Tomes I. II. 8vo. Vienne, 1790.

82. Cours de Chirurgie pratique fur la Maladie Venerienne, à l'Ufage des Eleves en Chirurgie; par M. *C. A. Lombard*, Maitre en Chirurgie de la Ville de Dôle, Departement du Jura; Chirurgien Major en Chef de l'Hopital militaire & auxiliaire de Strafbourg; ancien Chirurgien Major employé en cette Qualité à l'Armée de Corfe; Membre de plufieurs Academies, &c. 8vo. Strafbourg, 1790.

83. Flore des Environs de Paris, ou Diftribution methodique des Plantes qui y croiffent naturellement, executée d'apres le Syfteme de *Linnæus*, avec l'Indication du Temps de la Floraifon de chaque Plante; de la Couleur de fes Fleurs, & des Lieux où l'on trouve les Efpeces qui font moins communes. Par M. *Thuillier*, Botanifte. 8vo. Paris, 1790.

84. Precis

84. Precis sur la Canne, & sur les Moyens d'en extraire le Sel essentiel; suivi de plusieurs Observations sur le Sucre, sur le Vin de Canne, sur l'Indigo, sur les Habitations & sur l'Etat actuel de Saint Domingue: Ouvrage dedié a cette Colonie, & imprimé a ses Frais; par M. *Dutrone la Couture*, Docteur en Medecine, Associé de la Société Royale des Sciences & Arts du Cap François. 8vo. Paris, 1790.

85. Nouveau Plan de Constitution pour la Medecine en France; par la Société Royale de Medecine. 4to. Paris, 1790.

86. Vues generales sur la Restauration de l'Art de guerir, lues a la Seance publique de la Société de Medecine, le 31 Août, 1790, & presentées au Comité de Salubrité, de l'Assemblée Nationale, le 6 Octobre; suivies d'un Plan d'Hospices ruraux pour le Soulagement des Campagnes: par *Jean Gabriel Gallot*, Medecin de Montpellier, Membre de plusieurs Academies, Deputé de la ci devant Province de Poitou, Secretaire du Comité de Salubrité de l'Assemblée Nationale. 8vo. Paris, 1790.

87. Du Service des Hôpitaux militaires, rappélé aux vrais Principes; par M. *Coste*, premier Medecin des Camps & Armées du Roi. 8vo. Paris, 1790.

88. Nou-

88. Philosophische Botanic, mit critischen Bemerkungen; Erstes Band, von den mannigfaltigen Umhöeltungen der Samen; *i.e.* Philosophical Botany, with critical Observations; Vol. I. on the different Integuments of Seeds. By *Fred. Casimir Medicus*, M. D. Intendant of the Electoral Botanic Garden at Manheim. 8vo. Manheim, 1789.

89. Beobachtungen und Erfahrungen Medicinischen und Chirurgischen; *i. e.* Observations and Experiments Medical and Chirurgical. By *J. E. Trampel*, M. D. 2 Vols. 8vo. Lemgo, 1789.

90. Versuch über das Wechselfieber, und seine heilung besonders durch China Rinde; *i. e.* Essay on the Intermitting Fever, and particularly on its Cure by the Peruvian Bark. By *Frederick William Von Hoven*, Physician to the Duke of Wirtemberg, and to the City and Bailwick of Ludwigsburg. 8vo. Winterthur, 1789.

91. Medicinische und Chirurgische Bemerkungen; *i. e.* Medical and Chirurgical Observations. By *Maurice Gerhard Thilenius*, M. D. Physician to the City and Bailwick of Lauterbach. 8vo. Franckfort on the Mayne, 1789.

92. Che-

92. Chemische Zergliederung des Thuren-schen Wassers in Preussen; *i. e.* Chemical Analysis of the Water at Thorn in Prussia. By *K. C. Hagen.* 4to. Konigsberg, 1789.

93. Opere di *Ambrogio Bertrandi*, Professore di Chirurgia pratica nella R. Università di Torino, Membro della Reale Accademia di Chirurgia di Parigi, della Societa Reale di Torino, e primo Chirurgo della S. R. M. del fu Re Carlo Emanuele; pubblicate, e accresciute di note, e di Supplementi dai Chirurghi *Gio. Antonio Penchienati* e *Gioanni Brugnone*, Professori nella Regia Università, e Membri della Reale Accademia delle Scienze di Torino. Tom. VI. 8vo. Turin, 1786-8.

94. Ricettario Fiorentino nuovamente compilato e ridotto all' Uso moderno. 4to. Firenze, 1789.

95. Dei Bagni di Abano, trattato del Dott. *Salvat. Mandruzzato.* 4to. Padova, 1790.

96. Della Gonorrea virulenta, e della semplicita del medicare: Ragionamenti due di *Michel Angelo Roini.* 8vo. Napoli, 1790.

97. Dell' Antracite o Carbone di Cava detto volgarmente Carbon fossile; Compilazione fatta per Ordine del Governo. 8vo. Firenze, 1790.

INDEX.

### INDEX.

### A.

ABERDOUR, Alex. on the Small Pox, 207
Abfcefs, an epidemical Difpofition to, obferved, 114
Abforbents, the Inflammation of, after Wounds, oftentimes erroneoufly afcribed to poifonous Matter, — 115
Acid, dephlogifticated marine, its Effects, when applied to cancerous Ulcers, — — 200
——————— on animal and vegetable Subftances, — — 201
———————, adminiftered internally, — — — *ibid.*
———————, Caution with refpect to it, 202
Ackermann, J. C. G. Regimen Sanitatis Salerni, 215
Air, animal hepatic, its Properties defcribed, 198
—, atmofpheric, Differtation on, — 210
—, extricated from cancerous Matter and other animal Subftances, by Diftillation, Experiments relative to, 189
Aitken, Dr. John, on Fractures and Luxations, 203
Akenhead, Mr. his Accounts of the Effects of the Anguftura Bark, — — — 58, 66
Alexander, Gul. de partibus Corporis quæ viribus Opii parent, — — — 212
Amputation, fpeedy, in Cafes of compound Fracture of the Leg, Objections to, — — 8
Anatomy, Principles of, Expofition of, — 209
Anderfon, A. P. de Compofitione Acidi fulphurici, 212
————, Dr. James, Correfpondence relative to the Cochineal Infects, &c. — — 211
Aneurifm, fpontaneoufly cured, Cafe of, — 48
Anguftura Bark, Remarks on, —— 52
Animal Subftances, Obf. on the aërial Fluids extricated from, by Diftillation and Putrefaction, — 182
Anus, preternatural or artificial, Cafes of, 153, 163, 165
———————, Remarks on the Hiftory and Treatment of, —— —— 166
Aphrodifiacus, five de Lue Venerea, — 211
Apoplexy, Work relative to, — — 212
Apothecary's Mirror, — — 203

Apples Gaulis, M. d', Parallele entre les Mifericordes & les Hopitaux, — — 219

Afthma, fpafmodic, Differtation on, — 213

Auftin, Dr. W. on the Stone in the urinary Bladder, 208

B.

Bark, Anguftura. See Anguftura.
Baumes, M. fur les Malad. des Pays Marecageux, 218
Bell, Dr. J. on the Prevention of Difeafes in the Weft Indies, — — — 206
Bertrandi, Amb. Opere di, — — 224
Blagden, R. B. Cafe of Emphyfema, — 45
——— Aneurifm fpontaneoufly cured, 48
Blane, Dr. Gilbert, on Mufcular Motion, — 208
Blizard, William, on fome epidemical Effects, 105
Blood, Work relative to the Vitality of, — 207
Brougniart, M. his Account of a Cafe of Polydipfia, 85
Brouffonet, J. L. Victor, Corona Floræ Monfpel. 215
Bru, M. fur les Gateaux toniques, — 217
Burns and Scalds, Account of a Method of curing, 120

C.

Calculus. See Stone.
Camper, P. de fractura Patellæ & Olecrani, 212
Cancer, Matter of, Experiments and Obfervations on, 182
Cancerous Ulcers, peculiar Smell of, afcribed to a very volatile Subftance, — — 192
Carter, Henry Yates, Cafe of a compound Fracture of the Leg, — — — 1
——— Boy whofe Head was preffed between certain Parts of an Engine employed for draining a Coal Mine — — 11
——— Boy whofe left Leg and Thigh were torn off by a Mill, — 17
Catarrh, Differtation on, — — 213
Catheter, not to be introduced without great Care and Delicacy, — — — 117
———, Inftance of fatal Effects produced by it in inexperienced Hands, — — *ibid.*
Chalk, pounded or fcraped, recommended as an Application in certain Cafes of Burns and Scalds, — 124
Chaptal, M. Elements of Chemiftry, — 206, 220

Chaffagne,

Chaſſagne, M. Beſſejon de la, his Account of an Inſtance of exceſſive Thirſt, — — 74

Chauſſier, M. Obſ. Chirurgico-legales, - 219

Chemical Experiments and Opinions, extracted from a Work publiſhed in the laſt Century, - 203

——— and economical Eſſays, — 204

Chemiſtry, Elements of, — — 206, 220

———, Deſcription of a portable Cheſt of, 207

Cholera Morbus, Diſſertation on, — 210

Cleghorn, David, on the Treatment of Burns & Scalds, 120

Clidſdale, Jac. de Variolarum Contagionis Actione, 214

Coal, Italian Work relative to, — 224

Coleman, Edward, on ſuſpended Reſpiration, - 208

Collingwood, Dr. his Accounts of the Effects of the Anguſtura Bark, — — 55, 63

Collis, Edward, Diſſertation on Gonorrhœa, 207

Copper, Poiſon of, Work relative to, — 205

Corrie, Dr. James, on the Vitality of the Blood, 207

Coſte, M. du Service des Hopitaux militaires, 222

Cozens, W. R. on atmoſpheric Air, — 210

Crawford, Dr. Adair, on the Matter of Cancer, 182

Cynanche parotidea, Facts relative to it, — 113

D.

Delirium, an epidemical Diſpoſition to it in Caſes of Fracture, obſerved, — — 114

Default, M. on the Hiſtory and Treatment of preternatural Anus, — — — 153

Diagnoſis of Diſeaſes, Work relative to, — 207

Digeſtion of Food, Work relative to, — 209, 213

Digitalis purpurea. See Fox Glove.

Dodſworth, J. de Fluxu menſtruali & de Menorrhagia, 213

Durande, M. ſur les Pierres biliares, — 217

Dutrone la Couture, M. Precis ſur la Canne, 222

Dropſy, univerſal, Diſſertation on, — 210

Dyſentery, Works relative to, — 214, 217

Dyſpepſia, Diſſertation on, — — 213

E.

Earle, James, a Treatiſe on the Hydrocele, — 208
———, his Edition of Mr. Pott's Works, 202
Edwards, Dr. Geo. on the Diſeaſes of the human Body, 204

Effects, epidemical, Obfervations on, — 105
——————, with refpect to the human Body, hitherto not fufficiently attended to, — *ibid.*
——————, the Mode of conducting an Inquiry concerning them pointed out, — 106
——————, Advantages to be derived from a due Confideration of them, — — 117
Ehrhart, The. de Afphyxia Neonatorum, — 212
Electricity, Inftances of its good Effects in paralytic Affections, — — — 102
————, an erroneous Mode of applying it, in Cafes of mufcular Contraction, pointed out, — *ibid.*
————, medical, Work relative to, — 209
Emetics, Differtation on, — — 214
Emphyfema, brought on by Labour Pains, Cafe of, 45
Epidemical Effects. See Effects.
Eryfipelas, Inftances of, afcribed to epidemical Caufes, 112
Eftrade, M. l', fur les Pierres dans le Corps humain, 221
Eyes and Eyelids, Treatife on the Difeafes of, 204

F.

Fevers, Work relative to, — — 215
Fletcher, Jac. de Dyfpepfia, — — 213
Florence, Difpenfatory of, new Edition of, — 224
Ford, Mr. his Opinion with refpect to the fpontaneous Cure of Aneurifms corroborated, — 51
Fordyce, Dr. Geo. on the Digeftion of Food, 209
Foffils, Catalogue of a Collection of, — 221
Fothergill, Dr. Ant. Cautions to the Heads of Families, 205
Fox Glove, Differtation on, — — 214
Fracture, compound, of the Leg, Cafes of, — 1, 8
Fractures, Work relative to, — 203

G.

Gall Stones, Work relative to a fuppofed Solvent of, 217
Gallot, M. fur la Reftauration de l'Art de Guerir, 222
Gaterau, M. Defcription des Plantes de Montauban, 220
Gilby, Dr. William, on the Effects of Electricity in paralytic Affections, — — 102
Gifeke, Paul. D. Thefes Botanicæ, — 216
Goetz, M. Traité de la petite Verole & de l'Inoculation, 219
Gonorrhœa, Works relative to, — 207, 224

Gottling, J. F. A. Deſcription of a portable Cheſt of Chemiſtry, — — — *ibid.*

Gruner, C. G. Aphrodiſiacus, ſive de Lue Venerea, 211

H.

Harkneſs, G. de Alimentorum Concoctione, 213

Harries, Geo. de Vermibus Inteſtinorum, — *ibid.*

Head, Caſe of a remarkable Wound of, — 11

Hepatitis, Diſſertation on, — — 213

Hernia humoralis, obſerved to be ſometimes of general Occurrence, — — — 113

Horſes, Treatiſe on ſome of the Diſeaſes of, — 203

Hoſpitals, military, Work relative to, — 222

Hoſſack, David, on Cholera Morbus, — 210

Hoven, F. W. Von, über das Wechſelfieber, 223

Hugen, K. C. über das Thurenſchen Waſſer, 224

Hughes, T. Caſe of a fungous Enlargement of the Extremity of the female Urethra, — 26

Hunter, Oliv. de Catarrho a frigore, - 213

Hydrocele, Works relative to it, — 202, 208

Hyſteria, Diſſertation on, — — 214

I.

Inflammation, an epidemical Diſpoſition to it, obſerved, 114

——— of abſorbent Veſſels, after Wounds, oftentimes erroneouſly aſcribed to poiſonous Matter, 115

Inoculation of the Small Pox, Works relative to it, 207, 219

Intermitting Fever, Work relative to, — 223

K.

Ker, And. de Aſthmate ſpaſmodico, — 213

Kirkland, Dr. his Arguments againſt ſpeedy Amputation in certain caſes of Fracture, — — 10

Knapp, J. C. de Stimulis morboſis, — 211

L.

Lara, B. on the cuſtom of Mothers not ſuckling their Children, — — — 205

Lead, Poiſon of, Work relative to, — *ibid.*

Leber, Ferd. Eng. Tranſ. of his Prælect. Anatomicæ, 209

Leg, Caſes of a compound Fracture of, — 1, 8

Lewis, Dr. P. on common Water, — 203

Lithotomy,

Lithotomy, Work relative to it, — 218
Lombard, M. fur la Maladie Venerienne, — 221
Lowndes, Francis, on medical Electricity, — 209
Ludwig, C. F. Delectus Opufc. ad Scient. Nat. fpect. 215
Luxations, Work relative to, — — 203
Lyfimachia purpurea. See Scherbius.

M.

Maclean, Lachlanus, de Hepatitide, — 213
Maiden, W. his Account of a cafe of exceffive Thirft, 82
Mandruzzato, Salv. dei Bagni di Abano, — 224
Maxwell, G. his Account of a cafe of exceffive Thirft, 96
Medicus, F. C. Philofophifche Botanic, — 223
Menzies, Robertus, de Refpiratione, — 213
Merz, Joann. Jac. de Digitali purpurea, — 214
Meteorological Journals, their Utility, in certain Points of View, mentioned, — — 106
Metzger, J. D. Opufcula Anat. & Phyfiol. — 215
Montauban, Defcription of the Plants of, — 220
Monteggia, Bapt. Fafciculi Pathologici, — 212
Montpellier, Work relative to the botanical Hiftory of, 216
Mumps. See *Cynanche parotidea.*
Mufcular Motion, a Lecture on, — 208

N.

Necker, Nat. Jof. de, Elementa Botanica, — 216
Nicholfon, W. his Tranflation of M. Chaptal's Elements of Chemiftry, — — 206

O.

Ogle, T. on a Fact relative to the venereal Poifon, 209

P.

Paris, Work relative to the Plants in the Neighbourhood of, — — — 221
Pearfon, Dr. G. his Acc. of the Anguftura Bark quoted, 67
———, Samuel Burton, de Medicamentis vomitoriis, 214
Pennington, John, chemical and economical Effays, 204
Perinæum, Abfceffes of, Facts relative to, — 115
Perkins, Elijah, on univerfal Dropfy, — 210
Pharmacy, Work relative to the prefent State of, 203

Phyfic,

Phyfic, Plans relative to, under the new Conftitution of France, — — — 222
Polydipfia, Inftances of, — 73, 80, 82, 94
Pott, Percivall, his Arguments in favour of fpeedy Amputation, in certain cafes, objected to, — 9
——————, chirurgical Works of, — 202
Price, Philip Parry, on the Diagnofis and Prognofis of Difeafes, — — — 207
Proffer, Tho. on the Strangles and Fevers of Horfes, 203
Putrefaction, Procefs of, in the Lean of animal Subftances, — — — 195

R.

Raab, Mlle. de, Catalogue de fes Foffiles, — 221
Refpiration, fufpended, Works relative to, 208, 209
————, Differtation on, — — 213
Robertfon, Gul. de Hyfteria, — 214
Roini, M. A. della Gonorrea, — 224
Rowley, Dr. W. on the Difeafes of the Eyes, 204
Rufh, Dr. B. on the Effects of fpirituous Liquors, 210

S.

Salernum. See Ackermann.
Salkeld, Mr. his Account of the Effects of the Anguftura Bark, — — 67, 70, 71
Sanchéz, M. on the firft Appearance of the Venereal Difeafe in Europe, — — 202
Saucerotte, M. Hift. abregée de la Lithotomie, 220
Scalds, Account of a Method of curing. See Burns.
Scars, Obfervations relative to the Poffibility of eradicating them, — — 149
Scherbius, Joann. de Lyfimachiæ purp. virtute medica, 214
Schreger, B. G. de Irritabilitate Vaforum lymphat, 212
Skinner, Jofeph, his Tranflation of a Work by M. Sanchéz on the Hiftory of the Venereal Difeafe, — 202
Small Pox, Works relative to it, — 207, 214, 219
Sore Throat, malignant, Differtation on, — 214
Starrat, Joann. de Dyfenteria, — *ibid.*
Stimulants, their peculiar Effects accounted for, 109
Stone in the urinary Bladder, Works relative to, 208, 221
Strangles of Horfes, Treatife on, — 203
Sugar Cane, Work relative to it, — 222

Tenon,

T.

Tenon, M. his Account of two Inſtances of exceſſive Thirſt, — — — 80, 82

Thermometer, moral and phyſ. its Author aſcertained, 210

Thigh, Caſe in which it was torn off by a Mill, 17

Thilenius, M. G. Medicin. & Chirurg. bemerkungen, 223

Thirſt, exceſſive. See *Polydipſia.*

Thuillier, M. Flore des Environs de Paris, - 221

Tiſſot, M. ſur le Siege de la Pleureſie, — 219

Tode, Henr. J. de Fungis Mecklenburgenſibus ſelectis, 216

Trampel, J. E. Beobacht. und Erfahrungen Med. und Chirurg. — — — 223

V.

Vaughan, Walter, his Tranſ. of Leber's Prælect. Anat. 209

Venereal Diſeaſe, Works relative to it, 202, 207, 209, 217, 221, 224

Vinegar, recommended as an Application to Burns and Scalds, — — — 120

———, Directions with reſpect to the Mode of applying it, — — 121, 127, 147

———, its Effects in leſſening Pain and Inflammation in ſuch Caſes, — — — 122

———, in ſlight Caſes, is ſufficient to effect a Cure without any other Application, — — *ibid.*

———, what Sort of, is to be preferred for this Purpoſe, — — — 146

Vitman, Fulgent. Summa Plantarum, — 212

Vizaynus, Joſ. Pinille, de Febribus, — 215

Vogelius, S. G. de Submerſis, — — 214

Urethra, female, Caſe of a fungous Enlargement of the Extremity of, — — 26

W.

Wallis, Dr. Geo. his Annual Oration, — 203

Water, common, Inquiry into the Nature and Properties of, — — — *ibid.*

Waters, mineral, Works relative to, - 224

Weſt Indies, Work relative to the Prevention of Diſeaſes there, — — — 206

Wilkinſon, George, Remarks on the Anguſtura Bark, 52

Wilſon, Tho. de Cynanche maligna, — 214

Worms of the Inteſtines, Diſſertation on, - 213

Z.

Zulianius, Franciſcus, de Apoplexia, — 212

END OF THE SECOND VOLUME.

